# Syphilis and the Paretic Group

**Published:** 1910-08

**Authors:** Stewart R. Roberts

**Affiliations:** Atlanta, Ga.; Neurologist at the Wesley Memorial Hospital


### SYPHILIS AND THE PARETIC GROUP.

STEWART R. ROBERTS, M. S., M. D., ATLANTA, GA.

*Neurologist at the Wesley Memorial Hospital.*

Paresis is a disease which to be understood in all its phases must be considered as one of a group of related diseases of the nervous system having kinship with each other; and a maximal relation to syphilis as a cause. Locomotor ataxia, paresis, tabo-paresis and syphilitic insanity, constitute a group of four, closely related through a common cause. It is probable that there are occasional cases of paresis and locomotor ataxia not caused by syphilis, but the more we know of the nervous system, the two diseases and syphilis, the more evident becomes the fact that syphilis causes practically all these disease constituting the tabo-paretic group.

Since Fournier, in 1870, declared syphilis to be the cause of tabes, through much writing, many books, and more cases and experiments, the evidence has been accumulating that syphilis is the great cause of these once regarded separate diseases. They have been called para and meta-syphilitic conditions. They have been accused of being incurable diseases of long duration, responding but poorly to anti-syphilitic treatment. Ourselves ignorant until recently of the cause of syphilis, wrongly considering it arbitrarily caused, three staged, mysterious in its self-discovering and self-inflicting punishment, reappearing in the aparently cured individual a score of years later or in the next generation, how could we know what syphilis caused until we knew the cause of syphilis, and more wonderful still until we possess a definite scientific method of ascertaining whether a given individual ever had syphilis or not.

Through many years there has been coming home the apparently exaggerated statement of Osler that there were more people of a syphilitic taint in any community than people of a tubercular taint. For the first time in the history of medicine we are in a position to begin to study syphilis, and to study the problems centering in the effect of syphilis on the nervous system. First, the cause of syphilis is the mico-organism, spirochita pallida. This organism infects the nervous system and produces primarily an inflammation. (1) By round cell infiltration and exudation affecting chiefly the vessels of the cerebral membranes, and (2) by a parenchymatous degeneration of the cerebro-spinal axis or certain areas or tracts. Secondly, the discovery of the Wasserman reaction and the Noguchi reaction on blood serum. This enables us to discover definitely whether or not one has ever had syphilis, either in the acquired or hereditary form. These are as accurate as they are delicate, and while hardly possible except to the laboratory man, they have caused us to unlearn some of our neurology. As a recent editorial in the *Journal of the American Medical Association* stated: "Paresis is syphilitic insanity, and our so-called syphilitic insanity is but a stage of syphilis and a form of insanity. On a case recently one physician remarked the patient had either syphilitic insanity or paresis, it was hard to tell which. The patient had both, because there is no difference between them.

Francine discovered the Wassermann reaction in one patient 44 years and in another 33 years, after infection. McCampbell and Rowland found in 97.8 paretics a positive Wasserman and 95.6 positive with Noguchi's reaction.

Erb and his pupils found 95 per cent. of tabes with preceding syphilitic infection and Wassermann found 90 per cent. positive reaction in all tabes tested. Sachs could exclude syphilis as a cause in only one case in all his private practice. Clough found 7 cases of paresis, all showed a positive Wassermann reaction.

These tests prove:

1st. Once infected with syphilis, the infection is always demonstrable by this test.

2nd. One may have syphilis with no lesion that can be demonstrated and no history of infection. It is not always a disease with definite symptoms or with any symptoms.

3rd. Diseases of the nervous system due to syphilis are to be regarded not as separate diseases from the viewpoint of syphilis, but a tertiary or quartan manifestations of the disease. Wassermann's reaction when positive means active syphilis, and paresis is active syphilis of the brain. Tabes usually is active syphilis of the cord. The infection may be so profound as to involve both cord and brain, and then we have a tabo-paresis. Primary paresis usually involves the cord to a minor degree as seen in the occasional ataxias in these patients. Also primary tabes usually later involve the brain as shown by the ocular symptoms, or involvement of cerebral meninges and cortex.

A contrast of the symptoms is instructive in showing the similarity of the processes, remembering that both groups exist at the time in the same patient in tabo-paresis, and that syphilitic insanity is but a stage or species of paresis.

TABES.

1.—Lancilating pains.

2.—Argyll-Robertson pupil, optic atrophy.

3.—Loss of deep reflexes.

4.—Rhomberg symptom.

5.—Ataxia.

6.—Sexual weakness.

7.—Degeneration in cord, occasionally in brain also.

8.—Disease subject to remissions.
9.—Length of disease 1 to 15 years.

PARESIS.

1.—Premonitory mental symptoms.
2.—Immobility of pupil, often argyll-Robertson pupil.
3.—Changes in deep reflexes.
4.—Often headache, with occasional pains in lower extremities.
5.—More or less ataxia.
6.—Speech disturbanie.
7.—Muscle tremor.
9.—Rhomberg symptoms, occasional.
8.—Degeneration of cortex, occasionally in cord also.
10.—Diseases subject to remissions.
11.—Length of disease 1 to 15 years.

We have then the four diseases of the paretic group caused by syphilis, and being in reality syphilis in the fourth stage, or syphilis of the nervous system. In 1907, in the registration area in the United States, with a population of 32,000,000, there were 500,000 deaths; of these 2,116 were from paresis; 1,127 from tabes; 1 person in every 280 of the population of the eastern states and in England are insane, and in institutions for their care. In 1908, there were 5,301 first admissions for insanity in New York state, 664 of these, or 12 per cent., had paresis. In all New York state, there were only 1,368 deaths from typhoid; half as many people died from paresis in that state.

More people died of the disease in New York than from cancer of the breast or from angina-pectoris. One-eighth of all insanity is due to syphilitis, or to put it differently, if one-eighth of the insane had not had syphilis they would not be insane. The best way to cure syphilis is not to have it. Herbert Spencer believes the great development of the future will be a moral development, based on our highly developed nervous system. This much is certain, that the great strain of the present is on the nervous system, and in the future we may expect an ever increasing number of nerve deaths. The nervous system is the place of meeting of syphilis, wine, and worry, and the salvation of the nervous system is in nature, air and rest.

Take this case by Mendel: A man of 50, an alcoholic, marries his niece, aged 20. Three daughters were the fruit of the marriage, 11, 9, 7; all three epilepto-idiotic. Of the 5,301 New York cases, 654 were from alcoholic excesses, or were alcoholic psychoses; one-eighth more of all insane cases caused by alcohol and likewise preventable; one-fourth of all insanity is caused by alcohol and syphilis.

The human race has wondered at and admired the human mind. It is mysterious in its slow development, marvelous in its cunning, great in its accomplishment, and strange in its going. More mysterious and stranger still are the insane, the body left and the mind gone somewhere. Therefore it is no wonder we are late in coming to view the insane man as the definite effect of a definite cause, and the causes in one-fourth of all cases of insanity are syphilis and alcohol.

The insane man is to be pitied and cured if possible, but it is one disease which reacts on the family with the mark and measure of a social stigma. The family and friends lament what to them is a living death and worse.

As one walks through the asylums of this and other countries, and sees the wonderful care given and the great variety of these unfortunates, he unconsciously asks, will all this ever stop, and can any of it be decreased? A decrease of one-fourth is in our hands and in the hands of the people. The great paretic group would be no more but for syphilis, and alcoholic insanity is the last blow of dissipation on the brain. We come to view insanity not as mysterious in origin, but from definite causes, and as preventable, as in many cases, as typhoid or tuberculosis.

